# Comparison of the Anion Inhibition Profiles of the α-CA Isoforms (SpiCA1, SpiCA2 and SpiCA3) from the Scleractinian Coral *Stylophora pistillata*

**DOI:** 10.3390/ijms19072128

**Published:** 2018-07-21

**Authors:** Sonia Del Prete, Silvia Bua, Didier Zoccola, Fatmah A.S. Alasmary, Zeid AlOthman, Linah S. Alqahtani, Nathalie Techer, Claudiu T. Supuran, Sylvie Tambutté, Clemente Capasso

**Affiliations:** 1 Istituto di Bioscienze e Biorisorse, CNR, Via Pietro Castellino 111, 80131 Napoli, Italy; sonia.delprete@ibbr.cnr.it; 2 Dipartimento Neurofarba, Università degli Studi di Firenze, Sezione di Scienze Farmaceutiche e Nutraceutiche, Via U. Schiff 6, Sesto Fiorentino, 50019 Florence, Italy; silvia.bua@unifi.it (S.B.); claudiu.supuran@unifi.it (C.T.S.); 3 Department of Marine Biology, Centre Scientifique de Monaco, 8 Quai Antoine 1, 98000 Monaco, Monaco; zoccola@centrescientifique.mc (D.Z.); ntecher@centrescientifique.mc (N.T.); 4 Department of Chemistry, College of Science, King Saud University, P.O. Box 2455, Riyadh 11451, Saudi Arabia; fasmari@ksu.edu.sa (F.A.S.A); zaothman@KSU.EDU.SA (Z.A.); lAlqahtani@KSU.EDU.SA (L.S.A.); 5 Department of Chemistry, King Faisal University, Alahsa 31982, Saudi Arabia

**Keywords:** carbonic anhydrases, metalloenzymes, hydratase activity, anion inhibition profile, biomineralization, corals CAs, isoforms, recombinant enzyme

## Abstract

Carbonic anhydrases (CAs, EC 4.2.1.1) are widespread metalloenzymes used by living organisms to accelerate the CO_2_ hydration/dehydration reaction at rates dramatically high compared to the uncatalyzed reaction. These enzymes have different isoforms and homologues and can be found in the form of cytoplasmic, secreted or membrane-bound proteins. CAs play a role in numerous physiological processes including biomineralization and symbiosis, as is the case in reef-building corals. Previously, molecular and biochemical data have been obtained at the molecular level in the branching coral *Stylophora pistillata* for two coral isoforms which differ significantly in their catalytic activity and susceptibility to inhibition with anions and sulfonamides. More recently it has been determined that the genome of *S. pistillata* encodes for 16 CAs. Here, we cloned, expressed, purified and characterized a novel α-CA, named SpiCA3, which is cytoplasmic and ubiquitously expressed in all the cell layers including the calcifying cells. SpiCA3 is the most effective CA among the coral isoforms investigated and the most efficient catalyst known up to date in Metazoa. We also investigated the inhibition profiles of SpiCA3 and compared it with those obtained for the two other isoforms in the presence of inorganic anions and other small molecules known to interfere with metalloenzymes. These results suggest that *S. pistillata* has adapted its CA isoforms to achieve the physiological functions in different physicochemical microenvironments.

## 1. Introduction

The hydration/dehydration reaction of carbon dioxide (CO_2_ + H_2_O ⇌ HCO_3_^−^ + H^+^) is a crucial physiological reaction for the lifecycle of all living organisms since it is connected with numerous metabolic pathways, such as the biosynthetic processes requiring CO_2_ or HCO_3_^−^ (respiration, photosynthesis and carboxylation reactions) and biochemical pathways including pH homeostasis, secretion of electrolytes, transport of CO_2_ and bicarbonate, etc. [1,2,3,4,5]. The uncatalyzed hydration/dehydration has a rate too low (*k*_cat_ = 0.15 s^−1^ and *k*_-1_ = 50 s^−1^) for ensuring the appropriate levels of CO_2_ and HCO_3_^−^, which are substrates connected to the rapid aforementioned processes. Thus, living organisms use CAs to accelerate the CO_2_ hydration/dehydration reaction at rates dramatically high [3,4,5]. Generally, the *k*_cat_ of the CO_2_ hydration reaction is in the range of 10^4^–10^6^ s^−1^ [6,7,8]. Furthermore, it is not surprising in living organisms to find CAs with numerous isoforms and homologues and in the form of cytoplasmic, secreted or membrane-bound proteins [8,9,10,11,12]. The CA superfamily, in fact, represents a very interesting and complicated example of convergent/divergent evolution phenomena, with seven CA enzymatic families known to date, the α-, β-, γ-, δ-, ζ-, η-and θ-CAs [3,4,5]. Moreover, the majority of each class has multiple transcript variants and protein isoforms [3,4,5,10]. For example, 16 isoforms are known in vertebrates, all belonging to the α-class and with different subcellular localization: CAI, CAII, CAIII, CAVII, CA VIII, CA X, CA XI and CA XIII are found in the cytoplasm; CAIV, CAIX, CAXII, CAXIV and CAXV are the membrane-bound forms; CAVA and CAVB are the two mitochondrial forms; and the CAVI is the secretory form, [10,13]. The α-CAs are present in vertebrates, protozoa, algae, in the cytoplasm of green plants and in many Gram-negative bacteria; the β-CAs are widespread in both Gram-negative and positive bacteria, plants (mono- and dicotyledons) and fungi. The γ-CAs have been isolated in bacteria, cyanobacteria and Archaea, the δ-, ζ- and θ-CAs are present in marine diatoms, whereas the η-CAs are found in protozoa belonging to the *Plasmodium* spp. [14,15,16,17]. α-CAs are also the enzymatic machines used by invertebrates as well as vertebrates in the physiological process of biomineralization [18]. In the skeleton of corals (Cnidaria), in sea urchins tests (Echinoids), in molluscan shells, and in the eggshells of vertebrates, such as Reptilia and Aves, the mineral phase of the biogenic composite material is accumulated as calcium carbonate (CaCO_3_) under the polymorphs of aragonite and/or calcite [19]. CaCO_3_, together with silica, is one of the most known biogenic composites. Scleractinian reef-building corals are among the major biomineralizing organisms, which precipitate an aragonitic calcium carbonate skeleton. Pioneering studies conducted at the physiological level have shown that α-CAs are involved in coral biomineralization [20,21]. More recently, molecular and biochemical data have been obtained at the molecular level in the branching coral *Stylophora pistillata* [22]. In this species, 16 CAs isoforms have been found in the genome [9]. Among these, two α-CAs were isolated and named STPCA (renamed as SpiCA1) and STPCA-2 (renamed as SpiCA2) [9,23,24]. These CAs have been localized in the coral-calcifying cells, in the epithelium facing the skeleton and SpiCA2 is also present in the symbiotic endodermal cells. It has been proposed that SpiCA1 catalyzes the interconversion between the different inorganic forms of dissolved inorganic carbon at the site of calcification, while SpiCA2 is an intracellular enzyme, which is then found as an organic matrix protein incorporated in the skeleton [9,25]. Intriguingly, these CAs differ significantly in their catalytic activity and susceptibility to inhibition with anions and sulfonamides [21,24,26,27,28]. Here, we cloned, expressed, purified and characterized a novel α-CA, named SpiCA3, which is intracellular (cytoplasmic) and ubiquitously expressed in all the cell layers including the calcifying cells and the symbiotic endodermal cells. We also investigated the inhibition profiles of SpiCA3 and compared it with those obtained for SpiCA1 and SpiCA2 in the presence of inorganic anions and other small molecules known to interfere with metalloenzymes. These anions are known to bind to the CAs, but generally with less efficiency compared to the sulfonamides [4,29,30,31,32]. The inhibition study profiles together with the determination of the catalytic parameters of these enzymes are important for characterizing them and comparing their properties.

## 2. Results and Discussion

### 2.1. Enzyme Purification and Sequence Analysis

**Figure 1:**
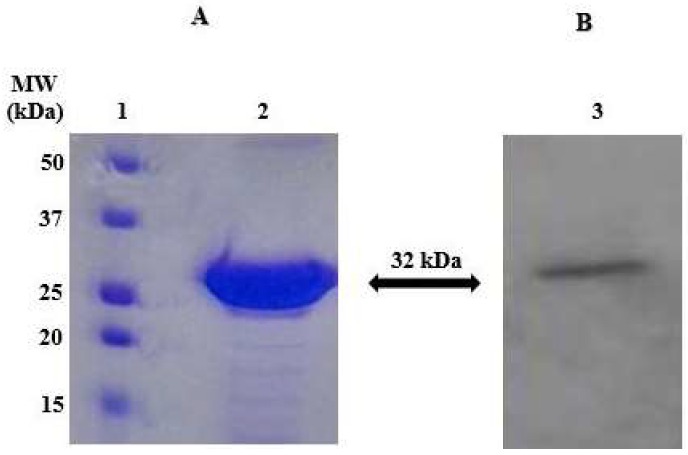
Sodium dodecyl sulfate (SDS)-polyacrylamide gel electrophoresis (PAGE) (Panel **A**) and Western blot (Panel **B**) of SpiCA3. Recombinant SpiCA3 was subjected to SDS-PAGE (Lane **2**, Panel **A**) and then electroblotted and incubated with anti-His Tag (Lane **3**, Panel **A**). Lane **1**, molecular markers (Panel **A**).

The recombinant SpiCA3 was produced as a fusion protein with a His-Tag tail in the cytoplasm of the *E. coli* BL21 DE3 cells. After sonication and centrifugation, most of the CA activity was recovered in the soluble fraction of cell extract. Using the affinity column (His-select HF Nickel affinity gel), SpiCA3 was purified to the homogeneity as a subunit with an apparent molecular weight of about 32 kDa as indicated by SDS-PAGE (Figure 1, Panel A) and Western blot analysis (Figure 1, Panel B). At this stage of the purification, SpiCA3 had a purity of 95% with a yield of 20 mg/3 g bacterial biomass (wet weight).

**Figure 2:**
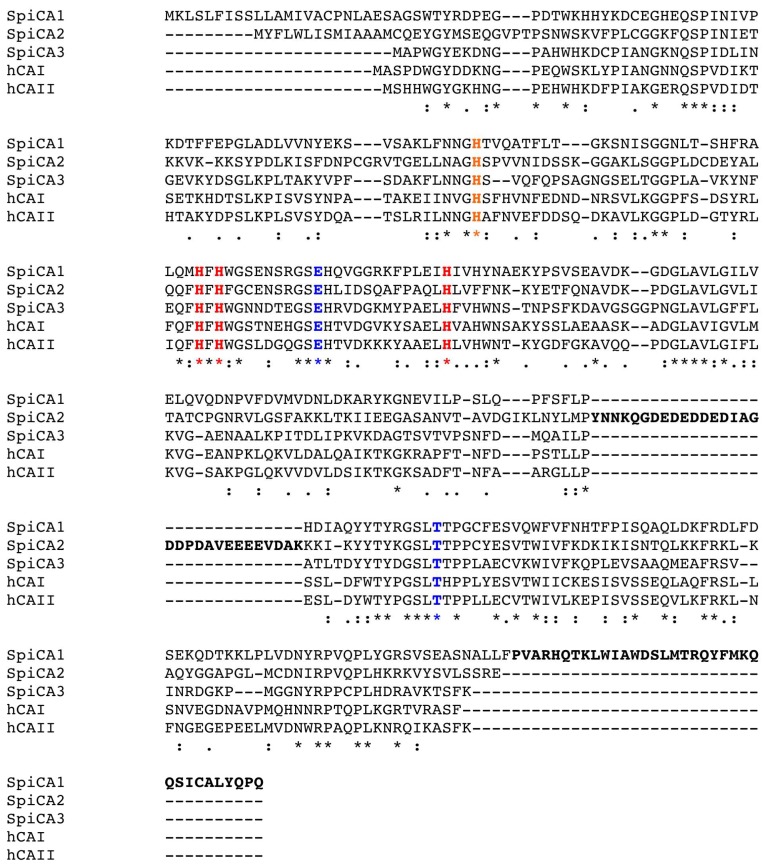
Multialignment of the α-CA amino acid sequences from human (hCAI and II) and *S. pistillata* (SpiCA1, SpiCA2 and SpiCA3). Multialignment was performed with the program Muscle. hCAI numbering system was used. Legend: zinc ligands, in red bold; “gate-keeper” residues, in blue bold; histidine proton shuttle, in orange bold; long stretches of 31 and 35 amino acid residues, in black bold; (*), indicates identity at a position; (:), designates conserved substitutions; (.) indicates semi-conserved substitutions; hCA I, *Homo sapiens*, isoform I (accession no. NP_001158302.1); hCA II, *Homo sapiens*, isoform II (accession no. AAH11949.1); SpiCA1, *S. pistillata* isoform 1 (accession no. ACA53457.1); SpiCA2, *S. pistillata* isoform 2 (accession no. EU532164.1); SpiCA3, *S. pistillata* isoform 3 (accession no. XP_022794253.1).

**Figure 3:**
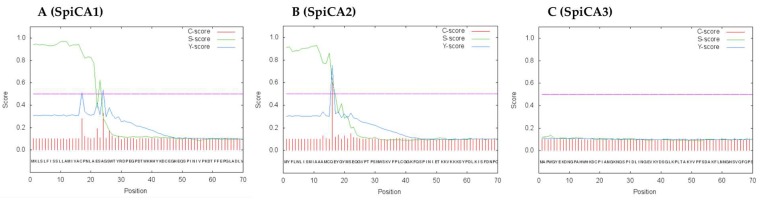
SignalP graphical output showing the three different scores C, S and Y, for the first 70 positions in the SpiCA1 (Panel **A**), SpiCA2 (Panel **B**), and SpiCA3 (Panel **C**) amino acid sequences. The program recognized the presence of a signal peptide at the N-terminal of the SpiCA1 and SpiCA2 amino acid sequences, but did not detect any signal peptide in SpiCA3. Legend: *X*-axis, amino acid position; C-score, raw cleavage site score; S-score, signal peptide score; Y-score, combined cleavage site score.

**Figure 4:**
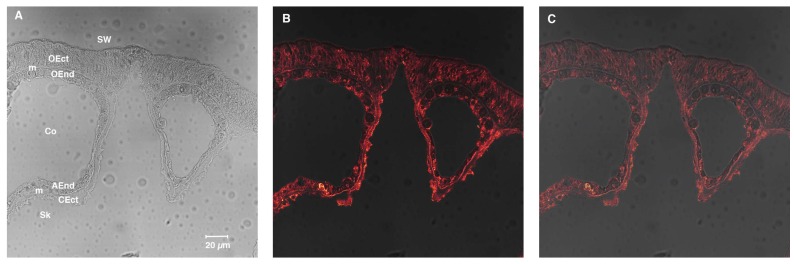
Immunolocalization of SpiCA3. Embedded cross-section of *Stylophora pistillata* tissues labeled by anti-SpiCA3. Panel (**A**) shows light transmission of the cross-section, (**B**) shows anti-SpiCA3 revealed by streptavidin AlexaFluor 568 secondary antibody that appears in orange (**C**) merges Panel (**A** and **B**). AEnd = Aboral Endoderm; CEct = Calcifying Ectoderm; Co = Coelenteron; m = Mesoglea; OEct = Oral Ectoderm; OEnd = Oral Endoderm; Sw = Seawater; Sk = Skeleton. The objective was 40× oil immersion.

SpiCA3, without the His-Tag, is a polypeptide chain of 258 amino acid residues with a theoretical molecular mass of 28.2 kDa (Figure 2). SpiCA3 was aligned with the amino acid sequences of SpiCA1 and SpiCA2, previously identified in *S. pistillata* and the two human isoforms, hCA I and II, in order to identify the presence of salient features of SpiCA3 (Figure 2). It may be observed that, like the other investigated α-CAs, SpiCA3 has the conserved three His ligands, which coordinate the Zn(II) ion crucial for catalysis, (His94, His96, and His119, hCA I numbering system). The proton shuttle residue (His64) is also conserved in all these enzymes. This residue assists the rate-determining step of the catalytic cycle, transferring a proton from the water coordinated to the Zn(II) ion to the environment. In this way, zinc hydroxide nucleophilic species of the enzyme is formed. SpiCA3 amino acid sequence had also the gate-keeping residues (Glu106 and Thr199), which orientate the substrate for catalysis and are also involved in the binding of inhibitors. The unique macro feature in the primary structure of the SpiCA3 with respect to the other two coral enzymes is the absence of a stretch of amino acids at its N-terminus. In fact, SpiCA1 and SpiCA2 are secreted metalloenzymes characterized by a signal peptide at the N-terminal of the amino acid sequence, which allows the protein translocation across the cytoplasmic membrane. Using the program SignalP 4.1 (http:// www.cbs.dtu.dk/services/SignalP/), a program able to identify the signal peptide at the N-terminus of the protein, it is readily apparent that SpiCA3 lacks the signal peptide (Figure 3, Panel C). Moreover, the SpiCA3 primary structure, like SpiCA1 and hCAI and II, showed a consistent deletion corresponding to a long stretch of 31 amino acid residues, which are present in the isoform SpiCA2 (Figure 2). But, similar to SpiCA2 and hCAI and II, in the amino acid sequence of SpiCA3 35 amino acid residues at the C-terminus, are missing too (Figure 2). The absence of these two long stretches of amino acid residues might significantly alter the three-dimensional structure of SpiCA3, determining significant differences in the protein catalytic activity as reported below (see Section 2.2). Such differences were in fact investigated in detail for α-CAs of bacterial and human origin [7,33], which also differ by the insertion of several loops, which leads to important differences of activity and sensibility to various classes of inhibitors. Sections of coral tissues were labeled with an anti-SpiCA3 antibody (see Section 4). When incubated with the pre-immune serum, no signal is observed (not shown) whereas with the antibody, all the tissues are labeled (Figure 4B) showing that SpiCA3 is ubiquitously present.

### 2.2. Kinetic Characterization

The kinetic parameters (*k*_cat_ and *k*_cat_/*K*_M_) for the SpiCA3 catalyzed CO_2_ hydration reaction are shown in Table 1. The kinetic constants were compared with those of the α-CAs from the *Homo sapiens* (isoforms hCAI and hCAII) as well as SpiCA1 and SpiCA2 previously identified in the genome of *S. pistillata*. SpiCA3 showed a *k*_cat_ of 1.6 × 10^6^ s^−1^ and a *k*_cat_/*K*_M_ of 1.5 × 10^8^ M^−1^ s^−1^. SpiCA3 resulted in the most effective CA among the coral isoforms investigated. It was 28.5- and 51.6-times more active as catalyst when compared with SpiCA2 and SpCA1, respectively (Table 1). Interestingly, SpiCA3 also showed a catalytic activity 1.14-times higher than hCA II, which is one of the most effective CO_2_ catalysts between the CAs known to date. Intriguingly, SpiCA3 was moderately inhibited by the clinically used sulfonamide CA inhibitor acetazolamide (AAZ), with an inhibition constant of 737 nM. The inhibition constants determined for SpiCA1 (*K*_I_ = 16 nM), SpCA2 (*K*_I_ = 74 nM), hCAII (*K*_I_ of 12 nM) and hCAI (*K*_I_ = 250 nM) were lower respect to the *K*_I_ of SpiCA3 (Table 1). From the kinetic parameters and the inhibition by acetazolamide, it can be concluded that SpiCA3 shows characteristics different from the coral enzymes, SpiCA 1 and SpiCA2. It is possible to speculate that the absence of the 31 and 35 amino acid residues in both the middle part and C-terminus of the SpiCA3 molecule, with respect to the other two coral enzymes, might be the key factor responsible for the different kinetic behavior of SpiCA3, making it one of the most efficient catalysts known to date in Metazoa (Table 1). The high catalytic efficiency of SpiCA3, together with its presence in symbiotic and calcifying cells, makes it a good candidate for supplying inorganic carbon for symbiosis and calcification.

### 2.3. Enzyme Protonography

**Figure 5:**
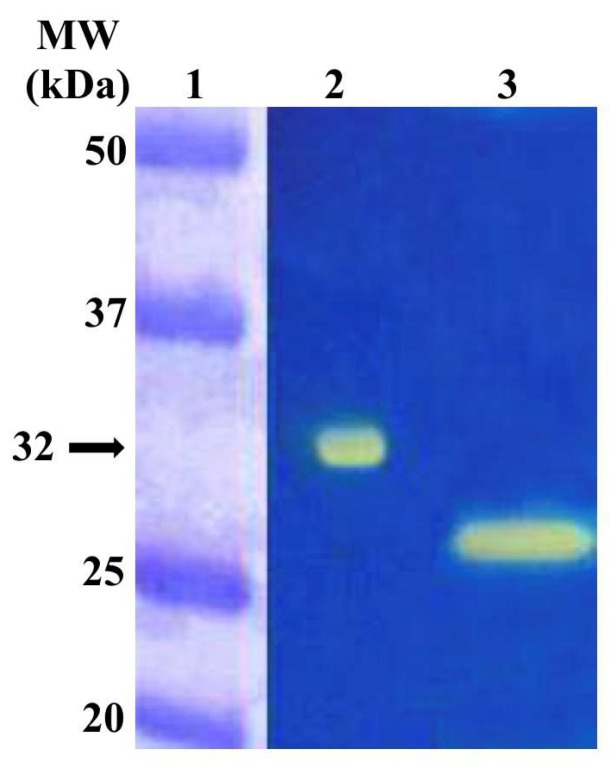
Protonography of the overexpressed recombinant SpiCA3 purified from the BL21 (DE3) cell extract using the His-select HF Nickel affinity column. The yellow band corresponds to the hydratase activity position on the gel. **Lane** 1, molecular markers; **Lane** 2, purified recombinant SpiCA3; **Lane** 3, commercial bovine CA used as positive control.

To investigate the hydratase activity of SpiCA3 on the acrylamide gel, samples of SpiCA3 were loaded on the gel at 10 µg/well. The protonography is based on monitoring the pH variation in the gel due to the CA-catalyzed conversion of CO_2_ to bicarbonate and protons. The production of ions (H^+^) during the CO_2_ hydration reaction can be visualized as a yellow band on the polyacrylamide gel. As can be observed in Figure 5, a yellow band on the gel corresponds to a monomer with an apparent molecular weight of 32 kDa. It is noteworthy that after removing SDS from the gel for developing the protonogram, the SpiCA3 monomer is able to correctly refold and generates the active enzyme, as demonstrated by the presence of the yellow band on the gel (Figure 5). This characteristic is also found in other CA-classes belonging to prokaryotic and eukaryotic organisms.

### 2.4. Inhibition with Anions

As inorganic anions constitute an important class of CA inhibitors (CAIs) [34], we have investigated the interaction of some of these compounds with SpiCA3 (Table 2). Indeed, information on the interaction of the coral CAs with anions might be crucial to design experiments and/or give insights relative to coral physiologic processes. The inhibition constants of the anions against SpiCA3 and other α-CAs, such as the two human isoforms hCAI and II, and the two *S. pistillata* isoforms, SpiCA1 and SpiCA2 are given in Table 2. From this table it can be noted that the most efficient inhibitors of SpiCA3 were sulfamide, diethylthiocarbamate, azide and cyanide, which showed a *K*_I_ in the range of 0.7–80 µM. Interestingly, sulfamide was also an efficient inhibitor of SpiCA1 (*K*_I_ = 10 µM) and SpiCA2 (*K*_I_ = 57 µM), but less efficient for hCAI (*K*_I_ = 310 µM) and hCAII (*K*_I_ = 1130 µM). Compared to human isoforms, poor inhibitory properties were also detected for the following anions: fluoride, chloride, bromide, iodide, cyanate, thiocyanate, bicarbonate, carbonate, nitrate, nitrite, hydrogensulfide, bisulfite, stannate, selenate, tetraborate, perruthenate, selenocyanate, trithiocarbonate, triflate, fluorosulfonate and iminodisulfonate, which showed inhibition constants in the range of 0.23–12.8 mM. Among these, bromide and iodide resulted in the most efficient inhibitor of SpiCA1 with a *K*_I_ = 9.0 and 9.7 µM. Bicarbonate, which is one of the substrates of the CAs, shows a similar *K*_I_ for the coral isoforms SpiCA1 (*K*_I_ = 450 µM) and SpiCA3 (*K*_I_ = 400 µM) and a very low affinity for SpiCA2 (*K*_I_ = 7.81 mM). Only the *K*_I_ of SpiCA2 is in the range of *K*_I_ observed for the human isoforms (*K*_I_ in the range of 12–85 mM). Concerning carbonate, the three coral isoforms show different characteristics. This inhibitor is 23.6- and 566-fold more effective for SpiCA2 (*K*_I_ = 240 µM) and SpiCA1 (*K*_I_ = 10 µM) respectively than for SpiCA3 (*K*_I_ = 5660 µM). Only the *K*_I_ of SpiCA3 is in the range of *K*_I_ observed for the human isoforms (*K*_I_ in the range of 15–73 mM).

Many other investigated anions did not significantly inhibit SpiCA3, such as tellurate, pyrophosphate, divanadate, perrhenate, peroxydisulfate, perchlorate, sulfamate, phenylboronic acid, phenylarsonic acid. All of them showed *K*_I_ > 100 mM against SpiCA3. Moreover, tetrafluoroborate and perchlorate showed a *K*_I_ > 200 mM against all the enzymes used in the present study. The inhibition pattern of the three coral enzymes shows how different the behavior of SpiCA1, SpiCA2 and SpiCA3 is towards these small anionic molecules. The dissimilar inhibition profile may be attributed to the different binding modes that each isoform has for the anions investigated, such as the different interactions of the amino acid residues and the metal ion at the active site with the inhibitors. For example, although anions were generally reported to directly coordinate to the metal ion form the enzyme active site [35,36], for a β-CA from the alga Coccomyxa [36], iodide was observed anchored to the zinc-coordinated water molecule, possessing thus a distinct inhibition mechanism. Such diverse inhibitory activity as the one observed here may in fact be ascribed to such putative, diverse interactions with the metal ion and its surrounding, but no detailed X-ray studies are available so far.

In the branching coral *Stylophora pistillata* the genome encodes for 16 CAs [9] but only three of them have been fully characterized for their chemical properties. Our results, which show that the sensitivity to anions varies between the three isoforms, suggest that the coral uses the CA isoforms to satisfy its physiological functions in different physicochemical microenvironments.

## 3. Conclusions

A novel α-CA, named SpiCA3 has been cloned, expressed, purified and characterized in the scleractinian coral *Stylophora pistillata*. This isoform is intracellular and ubiquitously expressed in all the cell layers of the coral including the symbiotic and calcifying cells. By comparing *k*_cat_/*K*_M_ values of Table 1, it can be observed that SpiCA3 is the most effective CA among the coral isoforms investigated, with a *k*_cat_ higher than that of the hCAII; this enzyme is thus the most efficient catalyst known to date in Metazoa. From a physiological aspect, this high catalytic efficiency makes SpiCA3 a good candidate for supplying inorganic carbon for symbiosis and calcification. The comparison of the anion inhibition profiles of SpiCA1, SpiCA2 and SpiCA3 shows that these three isoforms have a different behavior towards these small molecules. In particular, the most efficient inhibitors of SpiCA3 were sulfamide, diethylthiocarbamate, azide and cyanide, which showed a *K*_I_ in the range of 0.7–80 µM. Fluoride, chloride, bromide, iodide, cyanate, thiocyanate, bicarbonate, carbonate, nitrate, nitrite, hydrogensulfide, bisulfite, stannate, selenate, tetraborate, perruthenate, selenocyanate, trithiocarbonate, triflate, fluorosulfonate and iminodisulfonate were less efficient inhibitors with a *K*_I_ in the range of 0.23–12.8 mM. Our results showed that the sensitivity to anions and the catalytic properties differ between the three coral isoforms. The diverse inhibition profile is ascribed to the amino acid differences in the coral isoforms, which are responsible of the diverse interactions with the metal ion, as demonstrated for α-CAs of bacterial and human origin. Thus, the coral has adapted its isoforms to fulfill the physiological functions in different physicochemical microenvironments. Further work should address the other 13 isoforms found in the genome of *S. pistillata* in order to determine their properties and compare them together. The sensitivity of the different isoforms to inhibitors will help in the design of experiments aimed at better understanding what roles these isoforms play in coral physiological processes.

## 4. Materials and Methods

### 4.1. Chemistry

All salts/small molecules were of the highest purity available, from Sigma-Aldrich (Milan, Italy).

### 4.2. Gene Identification, Cloning, Expression and Purification

The gene of *S. pistillata* encoding for the α-CA SpiCA3 (NCBI Reference Sequence: XP_022794253.1) was identified running the “BLAST” program and using the nucleotide sequences of the coral α-CAs as query sequence, previously identified by our groups [9]. The GeneArt Company (Invitrogen, Waltham, MA, USA), specializing in gene synthesis, designed the synthetic gene (SpiCA3-DNA) encoding for a protein of 259 amino acid residues and containing four base-pair sequences (CACC) necessary for directional cloning at the 5′ end of the SpiCA3-DNA gene. The recovered SpiCA3-DNA gene and the linearized expression vector (pET-100/D-TOPO) were ligated by T4 DNA ligase to form the expression vector pET-100/SpiCA3. BL21 DE3 competent cells (Agilent) were transformed with pET-100/SpiCA3, grown at 37 °C and induced with 0.1 mM IPTG. After 30 min, ZnSO_4_ (0.5 mM) was added to the culture medium and cells were grown for an additional 4 h. Subsequently, cells were harvested and resuspended in the following buffer: 50 mM Tris/HCl, pH 8.0, 0.5 mM PMSF, and 1 mM benzamidine. Cells were then disrupted by sonication at 4 °C. After centrifugation at 12,000× *g* for 45 min, the supernatant was loaded onto a His-select HF Nickel affinity column (GE Healthcare, dimension: 1.0 × 10.0 cm). The column was equilibrated with 0.02 M phosphate buffer (pH 8.0) containing 0.01 M imidazole and 0.5 M KCl at a flow rate of 1.0 mL/min. The recombinant SpiCA3 was eluted from the column with 0.02 M phosphate buffer (pH 8.0) containing 0.5 M KCl and 0.3 M imidazole at a flow rate of 1.0 mL/min. Active fractions (0.5 mL) were collected and combined to a total volume of 2.5 mL. Collected fractions were dialyzed against 50 mM Tris/HCl, pH 8.3. At this stage of purification, the protein was at least 95% pure and the obtained recovery was of about 20 mg of the recombinant protein.

### 4.3. SDS-PAGE

Sodium dodecyl sulfate (SDS)-polyacrylamide gel electrophoresis (PAGE) was performed using 12% gels as described previously [39].

### 4.4. Western Blotting

The α-CA SpiCA3 was subjected to a 12% (*w*/*v*) SDS-PAGE, followed by electrophoretic transfer to a PVDF membrane with transfer buffer (25 mM Tris, 192 mM glycine, 20% methanol) using Trans-Plot SD Cell (Bio-Rad, Hercules, CA, USA). His-Tag Western blot was carried out using the Pierce Fast Western Blot Kit (Thermo Scientific, Waltham, MA, USA). Blotted membrane had been placed in the wash blot solution Fast Western 1 Wash Buffer to remove transfer buffer. Primary Antibody Working Dilution was added to the blot and incubated for 30 min at room temperature (RT) with shaking. Afterwards, the blot was removed from the primary antibody solution and incubated for 10 min with the Fast Western Optimised HRP Reagent Working Dilution. Subsequently, the membrane was washed two times in about 20 mL of Fast Western 1 Wash Buffer. Finally, the membrane was incubated with the detection reagent working solution and incubated for 1 min at room temperature and then developed with X-ray film.

### 4.5. Protonography

Wells of 12% SDS-gel were loaded with SpiCA3 or the commercial bovine CA (Sigma, St. Louis, Missouri, USA) mixed with Laemmli loading buffer containing SDS (1% final concentration), but without 2-mercaptoethanol. Samples were not boiled in order to avoid protein denaturation. The gel was run at 150 V until the dye front ran off the gel [40]. Following the electrophoresis, the 12% SDS-gel was subject to protonography to detect the SpiCA hydratase activity on the gel as described by Capasso et al. [41,42,43].

### 4.6. Immunolocalization with Anti-SpiCA3

Synthesis of polyclonal antibodies against SpiCA3 was produced in rabbit by Eurogentec (Liège, Belgium). Purified antibodies were raised against peptide NNDTEGSEHRVDGKM (amino acids 97–111) and peptide INRDGKPMGGNYRPP (amino acids 231–245). For immunolocalization, samples were prepared as previously described [44]. Briefly, apexes of *S. pistillata* were fixed in 3% paraformaldehyde in S22 buffer at 4 °C overnight and then decalcified using EDTA in CA-free S22 at 4 °C. They were then dehydrated in an ethanol series and embedded in Paraplast. Cross-sections (6 µm thick) were cut and mounted on silane-coated glass slides. Then, deparaffinized sections of tissues were incubated for 1 h in saturating medium. The samples were then incubated with the anti-SpiCA3 or the pre-immune serum as primary antibodies. After rinsing in saturating medium, samples were incubated with biotinylated anti-rabbit antibodies as secondary antibodies. After rinsing with PBS pH 7.4, samples were finally stained with streptavidin AlexaFluor 568. Samples were embedded in Pro-Long antifade medium (Molecular Probes) and observed with a confocal laser-scanning microscope (TCS SP5, Leica Microsytems CMS GmbH, Mannheim, Germany) equipped with visible laser lines. The excitation wavelength for Alexa Fluor 568 was 543 nm and the emission wavelength was 592–612 nm. The objective was 40× oil immersion.

### 4.7. Enzyme Kinetic and Inhibition

An Applied Photophysics stopped-flow instrument was used for assaying the CA-catalyzed CO_2_ hydration activity [37]. Phenol red (at a concentration of 0.2 mM) was used as indicator, working at the absorbance maximum of 557 nm, with 20 mM TRIS (pH 7.5) as buffer, and 20 mM NaClO_4_ (for maintaining constant the ionic strength), following the initial rates of the CA-catalyzed CO_2_ hydration reaction for a period of 10–100 s. The CO_2_ concentrations ranged from 1.7–17 mM for the determination of the kinetic parameters (by Lineweaver-Burk plots) and inhibition constants. For each inhibitor at least six traces of the initial 5–10% of the reaction have been used for determining the initial velocity. The uncatalyzed rates were determined in the same manner and subtracted from the total observed rates. Stock solutions of inhibitor (10–100 mM) were prepared in distilled-deionized water and dilutions up to 0.01 mM were done thereafter with the assay buffer. Inhibitor and enzyme solutions were preincubated together for 15 min at room temperature prior to assay, in order to allow for the formation of the E-I complex or for the eventual active site mediated hydrolysis of the inhibitor. The inhibition constants were obtained by nonlinear least-squares methods using PRISM 6 and the Cheng-Prusoff equation, as reported earlier [45,46,47], and represent the mean from at least three different determinations.

### 4.8. Primary Structure Analysis

Multialignment of amino acid sequences was performed using the program MUSCLE, a new computer program for creating multiple alignments of protein sequence [48].

## Figures and Tables

**Table 1:** Kinetic parameters for the CO_2_ hydration reaction catalyzed by the human cytosolic α-hCA isozymes I-II, and the coral enzymes SpiCA1-3, at 20 °C and pH 7.5 in 10 mM HEPES buffer, and their inhibition data with acetazolamide (5-acetamido-1,3,4-thiadiazole-2-sulfonamide), a clinically used drug.

Isozyme	Activity Level	*k*_cat_ (s^−1^)	*K*_cat_/*K*_M_ (M^−1^ s^−1^)	*K*_I_ (Acetazolamide) (nM)
hCAI	Moderate	2.0 × 10^5^	5.0 × 10^7^	250
hCAII	Very high	1.4 × 10^6^	1.5 × 10^8^	12
SpiCA1	Moderate	3.1 × 10^5^	4.6 × 10^7^	16
SpiCA2	High	5.6 × 10^5^	8.3 × 10^7^	74
SpiCA3	Very high	1.6 × 10^6^	1.5 × 10^8^	737

**Table 2:** Inhibition constants of anionic inhibitors against isozymes hCA I, II and VI (human, α-CA class enzymes), and the CA from the coral *Stylophora pistillata*, SpiCA1–STPCA3, for the CO_2_ hydration reaction, at 20 °C [37].

Inhibitor	*K*_I_ [mM] ^#^				
	hCA I ^a^	hCA II ^a^	SpiCA1 ^b^	SpiCA2 ^b^	SpiCA3 ^c^
F^−^	>300	>300	0.62	0.92	0.48
Cl^−^	6	200	0.50	0.53	0.51
Br^−^	4	63	0.0097	0.96	0.23
I^−^	0.3	26	0.0090	33.0	0.56
CNO^−^	0.0007	0.03	0.59	0.69	2.41
SCN^−^	0.2	1.6	0.68	0.51	2.53
CN^−^	0.0005	0.02	0.58	0.86	0.050
N_3_^−^	0.0012	1.5	0.52	4.68	0.080
HCO_3_^−^	12	85	0.45	7.81	0.40
CO_3_^2−^	15	73	0.010	0.24	5.66
NO_3_^−^	7	35	0.56	0.99	12.8
NO_2_^−^	8.4	63	0.77	3.15	0.45
HS^−^	0.0006	0.04	0.58	3.94	0.34
HSO_3_^−^	18	89	0.41	0.43	5.20
SO_4_^2−^	63	>200	0.91	0.33	0.61
SnO_3_^2−^	0.57	0.83	nt	nt	2.96
SeO_4_^2−^	118	112	nt	nt	5.14
TeO_4_^2−^	0.66	0.92	nt	nt	>100
P_2_O_7_^4−^	25.8	48.5	nt	nt	>100
V_2_O_7_^4−^	0.54	0.57	nt	nt	>100
B_4_O_7_^2−^	0.64	0.95	nt	nt	0.84
ReO_4_^−^	0.11	0.75	nt	nt	>100
RuO_4_	0.10	0.69	nt	nt	0.76
S_2_O_8_^2−^	0.11	0.084	nt	nt	>100
SeCN^−^	0.085	0.086	nt	nt	0.15
CS_3_^2^^−^	0.0087	0.0088	nt	nt	0.47
Et_2_NCS_2_^−^	0.00079	3.1	nt	nt	0.044
Triflate	nt	nt	nt	nt	0.29
BF_4_^−^	>200	>200	>200	>200	>200
ClO_4_^−^	>200	>200	>200	>200	>200
FSO_3_^−^	nt	nt	nt	nt	0.55
NH(SO_3_)_2_^2−^	nt	0.76	nt	nt	0.48
H_2_NSO_2_NH_2_	0.31	1.13	0.010	0.057	0.0007
H_2_NSO_3_H	0.021	0.39	0.81	0.085	>100
Ph-B(OH)_2_	58.6	23.1	0.68	0.081	>100
Ph-AsO_3_H_2_	31.7	49.2	0.78	0.067	>100

^#^ Mean from three different assays. ^a^ Human recombinant isozyme, data from Reference [38] ^b^ Coral recombinant enzyme, data from Reference [26,27]. ^c^ This work. nt = not tested.
